# Hepatitis B virus X protein amplifies TGF-β promotion on HCC motility through down-regulating PPM1a

**DOI:** 10.18632/oncotarget.8884

**Published:** 2016-04-21

**Authors:** Yuan Liu, Yong Xu, Hongxin Ma, Bo Wang, Leiqi Xu, Hualin Zhang, Xiaojia Song, Lifen Gao, Xiaohong Liang, Chunhong Ma

**Affiliations:** ^1^ Key Laboratory for Experimental Teratology of Ministry of Education and Department of Immunology, Shandong University School of Medicine, Jinan, Shandong, 250012 P.R. China

**Keywords:** HCC, HBx, PPM1a, TGF-β, tumorigenenesis

## Abstract

Over-activation of transforming growth factor-β (TGF-β) signaling pathway promotes cell migration and invasion in hepatocellular carcinoma (HCC). The Hepatitis B virus X protein (HBx) is involved in the enhancement of TGF-β signaling pathway in HCC while the mechanism remains unclear. Protein phosphatase magnesium dependent 1A (PPM1a) functions as a phosphatase essential for terminating the TGF-β signaling pathway by dephosphorylating p-Smad2/3. In this study, we found that HBx dose-dependently downregulated PPM1a protein level in the presence of TGF-β, while having no effect on its mRNA level. Further study showed that HBx increased the ubiquitination of PPM1a and accelerated its proteasomal degradation. Restoration of PPM1a almost completely abrogated HBx mediated promotion on HCC migration and invasion. This involvement of PPM1a in HBx-related HCC was further confirmed with immunohistochemical analysis in HCC tissue. Compared with paired pericarcinous tissue, HCC tissue showed decreased PPM1a level. Besides, PPM1a level is negatively correlated with HBx expression. Taken together, our present study suggests that HBx-induced degradation of PPM1a is a novel mechanism for over-activation of TGF-β pathway in HCC development, which might provide potential candidates for clinical diagnosis and treatment.

## INTRODUCTION

Nowadays, primary liver cancer has become the fifth most common cancer and the third most common cause leading to cancer mortality in the world, and an approximated 1 million new liver cancer cases occur on a global scale annually [1, 2]. About half of these cases occur in China [3]. Hepatocellular carcinoma (HCC) accounts for 70% to 85% of primary liver cancers [4] and Hepatitis B Virus (HBV) infection remains the most frequent underlying cause of HCC in the world [2, 5]. Hepatitis B virus X protein (HBx), a regulatory viral protein, has been reported to play complicated roles in hepatocarcinogenesis via different mechanisms [6].

Transforming growth factor-β (TGF-β), a 25 kDa cytokine, is frequently over-expressed in tumors including HCC [7, 8]. Accumulated data showed that TGF-β plays double-edged roles in HCC, working as a tumor suppressor to inhibit cell growth at early stages of liver damage and regeneration, while as a tumor promoter to induce epithelial-mesenchymal transition (EMT) and enhance cancer metastasis and invasion in advanced HCC [9]. TGF-β exerts its function through its downstream signaling pathway. It is initiated by binding of TGF-β with TGF-β receptor type-2 (TGFBR2), which recruits and catalyzes the phosphorylation of type 1 receptor as a serine/threonine kinase. Type 1 receptor in turn phosphorylates Smad2 and Smad3 in the cytoplasm. Then, with the assistance of Smad4, phosphorylated Smad2 and Smad3 are transported into the nucleus, where they cooperate with specific transcription factors to regulate gene transcription [10].

Actually, cancer cells tend to be refractory to the tumor suppressive activity of TGF-β, while still remain sensitive to its tumor-promotion effect [11]. Besides, not only up-regulated TGF-β expression but also enhanced Smad2/3 phosphorylation is observed in HCC, which benefits for tumor development. Therefore, TGF-β and its downstream signaling molecules have become a pharmacological target in liver cancer [12], and to uncover the regulatory mechanism of TGF-β pathway is of great importance.

Studies have shown that HBV and its viral proteins are at least partially responsible for over-activation of TGF-β signaling in HBV-related HCC [13, 14]. Lee et al found that HBx enhanced phosphorylation of pSmad2/3C by binding with Smad4 and amplified TGF-β signal pathway in several HCC cell lines, which led to the enhanced transcriptional activation of TGF-β-responsive genes [15]. To probe into the interaction between HBV and TGF-β would be beneficial for the discovery of therapeutic drug targeting to TGF-β signal pathway in HBV-related diseases.

Protein Phosphatase Magnesium Dependent 1A (PPM1a), also named PP2Cα, is a phosphatase belonging to PP2C class [16]. It was identified as the only phosphatase responsible for dephosphorylation of p-Smad2/3, answering the long-standing question how TGF-β signal pathway terminates in the presence of continuous TGF-β [17]. Recently, PPM1a/PP2Cα was found to play a role in wound healing [18] and tumor metastasis [19, 20] by inhibiting TGF-β signal pathway. Importantly, several tumor tissues [19, 20], including HCC [21], showed decreased or loss of PPM1a expression, indicating that uncovering the regulatory mechanisms of PPM1a expression might be useful for explaining the aberrant status of TGF-β signal pathway in tumor development.

Here we found that on the stimulation of TGF-β, HBx represses PPM1a expression in a ubiquitin-dependent protein degradation pathway, and the decreased PPM1a expression is responsible for HBx-induced promotion on cell mobility. Rescue of PPM1a expression almost abrogates HBx-enhanced HCC metastasis in vitro, indicating that the suppressing of PPM1a by HBx may be a novel mechanism of HCC carcinogenesis. Moreover, human HCC tissue also showed a negative correlation between PPM1a and HBx level. Our data here might shed new light on the regulation of TGF-β signal pathway and provide new evidence for PPM1a as the potential target in HCC chemotherapy.

## RESULTS

### HBx down-regulates PPM1a protein level on TGF-β stimulation

**Figure 1:**
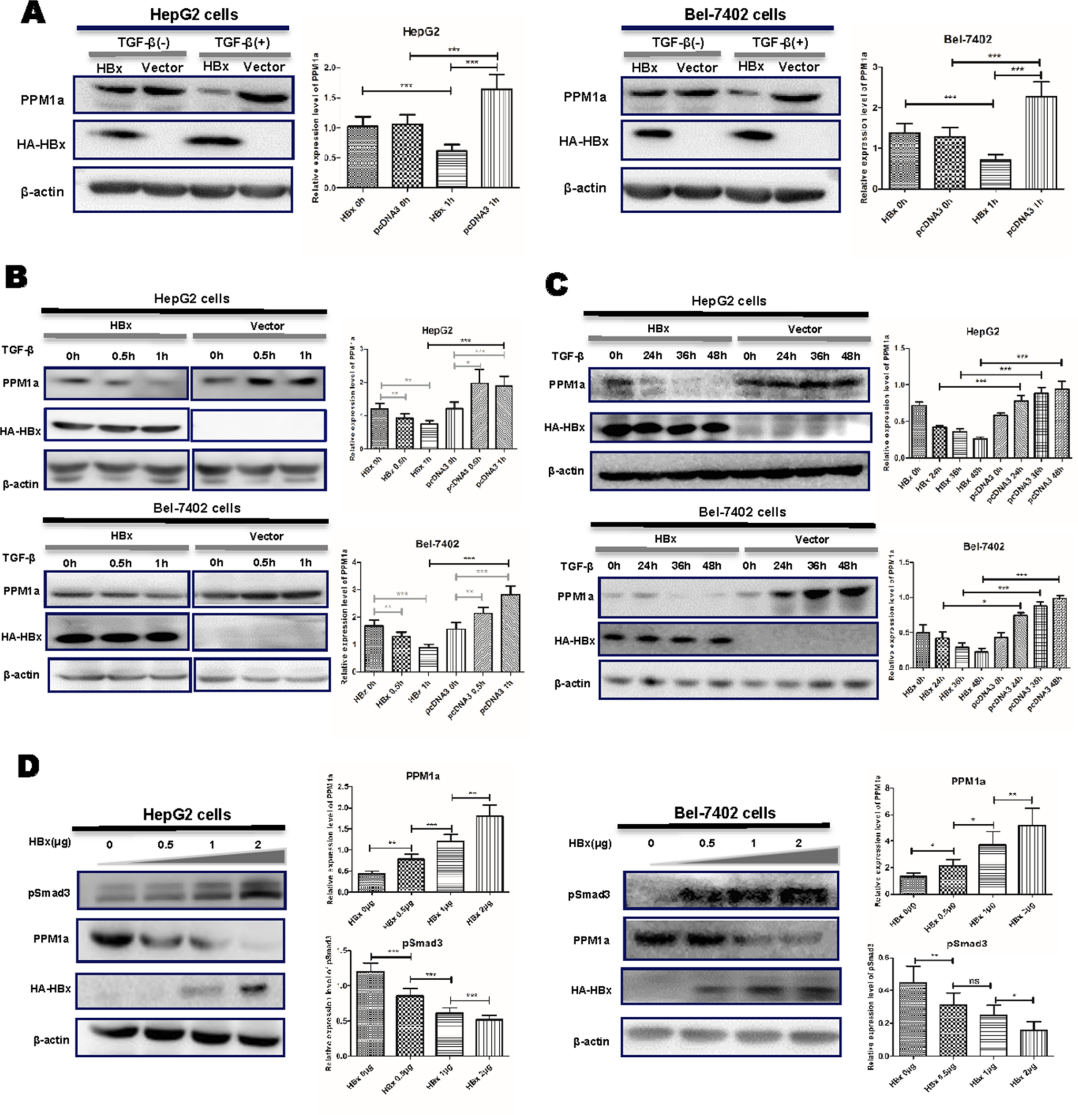
HBx downregulated PPM1a expression in the presence of TGF-β HepG2 and Bel-7402 cells were transfected with pcDNA3-HBx-HA or pcDNA3 vector for 48 h following with no further treatment or following with the treatment of TGF-β for 1h **(A).** HepG2 and Bel-7402 cells were transfected with pcDNA3-HBx-HA or pcDNA3 vector and then treated with TGF-β for 0, 0.5 and 1h **(B).** or 24, 36 and 48h **(C).** HepG2 and Bel-7402 cells were transfected with 0, 0.5, 1 and 2.0μg of pcDNA3-HBx-HA and then stimulated with TGF-β for 1h **(D).** Expression of PPM1a and HBx was measured by Western blot. Band intensity of each Western Blot image is analyzed by the software Image J. The quantitative ratios are presented as relative optical densities of bands that are normalized to the level of actin. The data are represented as the mean ± SD from at least three independent experiments. ns p>0.05, * 0.01<p<0.05, **0.001<p<0.01, *** p<0.001 as evaluated using Student's t test.

To investigate the effect of HBx on PPM1a expression, we transiently transfected HBx expression plasmid pcDNA3-HBx-HA into HepG2 or Bel-7402 cells. After transfection for 48 hrs, total protein was extracted and the expression of the PPM1a protein was detected. There was no detectable difference in PPM1a protein level between HBx-transfected cells and pcDNA3 control cells. However, when treated with 5ng/ml of TGF-β, PPM1a expression was significantly down-regulated by HBx in both HCC cell lines (Figure 1A). Besides, consistent with reported data about the feedback up-regulation of PPM1a expression on TGF-β stimulation, pcDNA3-transfected group showed significantly increased PPM1a protein upon short TGF-β stimulation (30min) (Figure 1B), and this effect last until 48h after treatment (Figure 1C). However, with the presence of HBx, PPM1a instead decreased dramatically after exposure to TGF-β in a time-dependent manner and still remained at lower level than that in pcDNA3 control cells until 48h after stimulation (Figure 1B, 1C). Furthermore, the inhibition of PPM1a by HBx is also in a dose-dependent fashion (Figure 1D). Consistent with the role of PPM1a as the negative regulator of TGF-β signaling, HBx dose-dependently upregulated pSmad3 level which represent the activation of TGF-β signaling (Figure 1D), indicating that HBx may amplify TGF-β signaling through down-regulating PPM1a. Same results were got in HepG2 and Bel-7402 cells transfected with pcDNA3-HBV1.1 which contains 1.1 copy of HBV genome and can mediated the expression of all HBV encoded proteins [22] (Supplementary Figure 1). These data clearly showed that HBx decreased PPM1a protein expression in the context of TGF-β stimulation, which might be a novel mechanism for the repressed expression of PPM1a in HCC tissue.

### HBx and TGF-β have synergistic effect on promoting HCC metastasis

**Figure 2:**
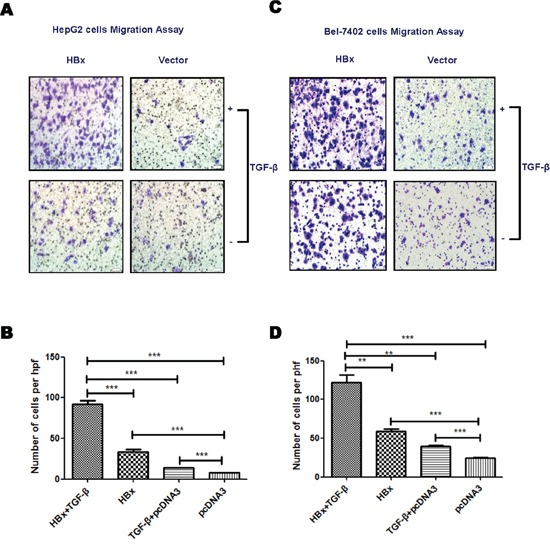
HBx enhances TGF-β stimulation on cell mobility HepG2 or Bel-7402 cells transfected with pcDNA3-HBx-HA or pcDNA3 were treated with TGF-β (5ng/ml) or PBS. Transwell assays was performed to analyze the migration ability of HepG2 **(A** and **B).** and Bel-7402 **(C** and **D).** cells. Representative images for migration assay were shown (×200). Statistical analysis of each group is shown as Mean ± SD from three independent experiments. ns p>0.05, * 0.01<p<0.05, **0.001<p<0.01, *** p<0.001. Quantification of cell migration and invasion in results represents cell counts from 5 randomly selected low-powered fields (×200).

As HBx down-regulates PPM1a and amplifies phosphorylation of Smad3, we hypothesized that HBx and TGF-β may have synergistic effect on promoting HCC metastasis. In order to address this, transwell assay was performed with HBx-overexpressed HepG2 and Bel-7402 cells exposed to 5ng/ml of TGF-β. As shown in Figure 2A and 2B, HBx enhanced the migration of HepG2 cells, which was further augmented by TGF-β stimulation. This synergetic effect of HBx and TGF-β on cell migration was also found in Bel-7402 cells (Figure 2C, 2D). These data supported that HBx enhanced the migration of HepG2 cells, which was further increased by TGF-β stimulation.

### PPM1a overexpression reverses HBx-mediated enhancement on cell mobility

**Figure 3:**
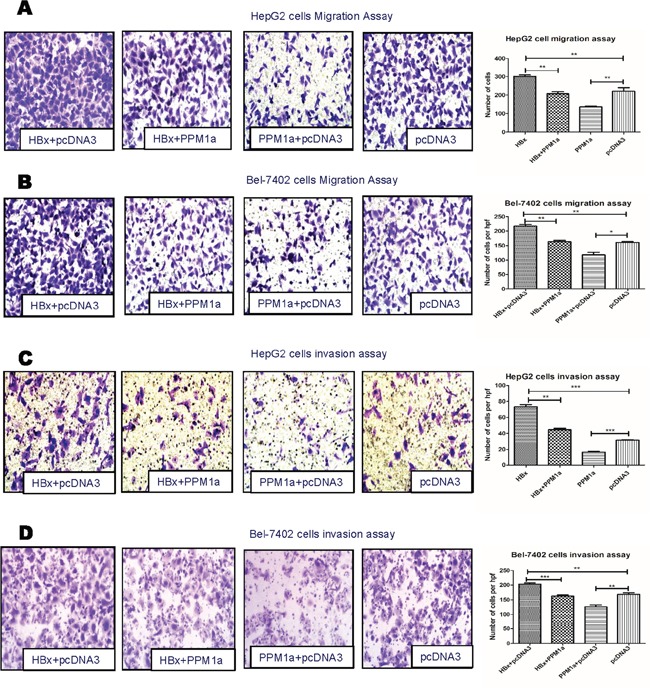
PPM1a overexpression abrogates HBx-enhanced cell mobility HepG2 cells **(A** and **C).** and Bel-7402 cells **(B** and **D).** were transfected with 1μg of pcDNA3-HBx-HA and/or pcDNA3-PPM1a-Flag with continuous TGF-β stimulation (5ng/ml). Cell mobility was detected by migration assay **(A** and **B)** and invasion assay **(C** and **D)**. All the experiments in Figure 3 have been done upon continuous TGF-β treatment (5ng/ml). Representative images for migration and invasion assay were shown in left panel (× 200). Statistical analysis of each group is shown as Mean ± SD. ns p>0.05, * 0.01<p<0.05, **0.001<p<0.01, *** p<0.001. Quantification of cell migration and invasion in results represents cell counts from 5 randomly selected low-powered fields (×200).

In order to test whether down-regulating PPM1a protein is involved in HBx-induced promotion on HCC metastasis, we restored the level of PPM1a in cells expressing HBx and then measured cell migration and invasion. As shown in Supplementary Figure 2, transfection of 1μg of pcDNA3-PPM1a-Flag almost leveled PPM1a expression of HBx-transfected cells to that of control cells. Moreover, transwell migration and invasion assays showed that while HBx alone enhanced cell mobility and invasiveness, co-transfection of HBx and PPM1a almost reversed this promotion effect in both HepG2 and Bel-7402 cells (Figure 3). Considering that HCC patients tend to have an elevated expression of TGF-β [23, 24], these results indicated that down-regulation of PPM1a with the presence of TGF-β may be a novel mechanism by which HBx promotes hepatoma cell migration and invasion.

### HBx induces ubiquitin-mediated proteasomal degradation of PPM1a

**Figure 4:**
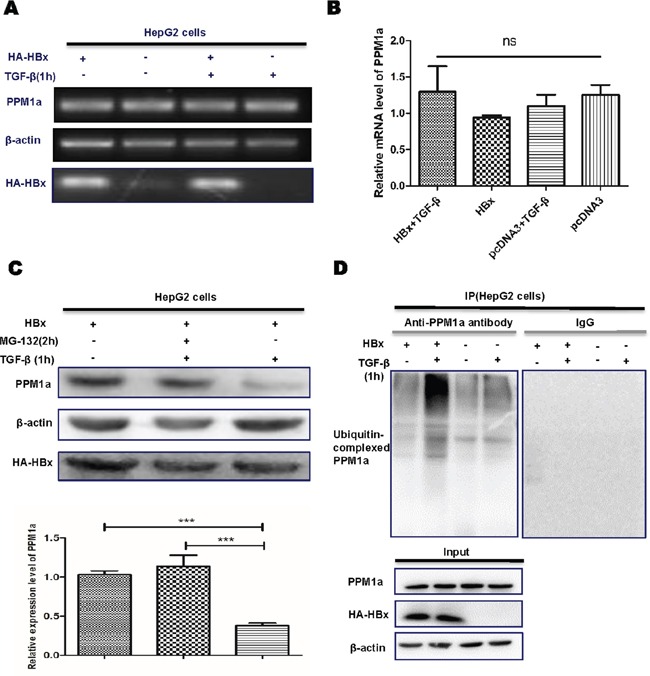
HBx accelerates ubiquitination and proteasomal-mediated degradation of PPM1a **A.** and **B.** The pcDNA3-HBx-HA or pcDNA3 were transfected into HepG2 cells, with or without 1h of TGF-β stimulation. PPM1a mRNA level were determined by **(A)** RT-PCR or **(B)** realtime PCR. **C.** HepG2 cells transiently transfected with pcDNA3-HBx-HA were treated with 100 mM MG132 or DMSO for 2 h before harvesting with or without TGF-βstimulation for 1h and level of PPM1a was measured by Western Blot. Band intensity of each Western Blot image is analyzed by the software Image J. The quantitative ratios are presented as relative optical densities of bands that are normalized to the level of actin. **D.** pcDNA3-HBx-HA or pcDNA3 were transfected into HepG2 cells with or without 1h of TGF-β stimulation. Immunoprecipitation assay was performed with anti-PPM1a antibody and levels of ubiquitin-conjugated PPM1a were detected with anti-ubiquitin antibody by western blot.

Next, we tried to explore how HBx down-regulates PPM1a in hepatoma cells. As shown in Figure 4A and 4B, HBx had no obvious effect on PPM1a mRNA level in HepG2 cells. Previous studies disclosed that activities of type2C protein phosphatases are finely regulated by their protein degradation to a great extent [25]. Thus, we examined the effect of HBx on PPM1a protein degradation. Results showed that the effect of HBx on PPM1a protein level was almost completely abolished when cells were treated with the proteasome inhibitor MG132 (Figure 4C), indicating that HBx may affect the proteasomal degradation of PPM1a. As ubiquitination is generally enhanced preceding proteasomal degradation [26], we then examined whether HBx enhanced ubiquitination of PPM1a. Results of immunoprecipitation assay demonstrated that, after TGF-β (5ng/ml) was added for 1h, it seemed that the amount of ubiquitin-complexed PPM1a slightly decreased in pcDNA3-transfected group, consistent with the upregulated PPM1a level upon TGF-β stimulation. However, when HBx was overexpressed in HepG2 cells treated with TGF-β, ubiquitination of PPM1a was significantly enhanced (Figure 4D). Taken together, these results indicated that HBx downregulates PPM1a protein level by inducing its ubiquitin-dependent proteasomal degradation, which may be a novel mechanism of TGF-β over-activation during HCC development.

### Negative correlation between PPM1a and HBx in HCC samples

**Figure 5:**
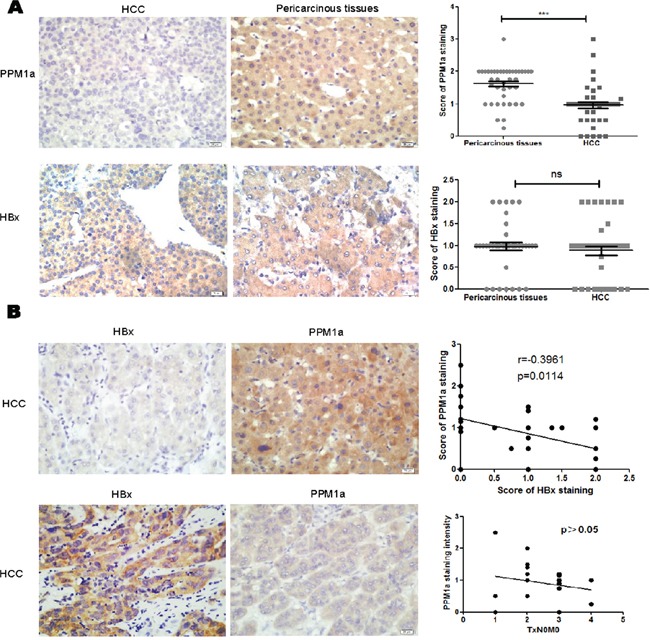
Negative correlation between HBx and PPM1a expression in HCC tissue **A.** Expression of PPM1a and HBx in HCC tissue and pericarcinous tissue was detected by immunohistochemistry (IHC). Original magnification 400× (left panel). Statistical analysis of PPM1a and HBx expression intensity in HCC tumor tissue and adjacent non-tumor tissue were shown in right panel. **B.** Expression of PPM1a and HBx level were assessed in HCC tissue by IHC. Original magnification 400× (left panel). Correlation between PPM1a and HBx expression intensity and between PPM1a and T stage were analyzed statistically (right panel). ns p>0.05, * 0.01<p<0.05, **0.001<p<0.01, *** p<0.001.

To further confirm the regulation of HBx on PPM1a, we also analyzed the relationship between HBx and PPM1a expression in HBV-related HCC specimens. First, our data showed that PPM1a expression is negatively associated with pSmad3 staining intensity, suggesting that PPM1a here acts as a functional phosphatase terminating TGF-β smad signal pathway (Supplementary Figure 3). Second, as shown in Figure 5A, PPM1a staining was mostly localized in the cytoplasm of pericarcinous liver tissue and HCC tissue, while its expression in HCC tissue was dramatically weaker than that in paired surrounding non-cancerous tissue (P < 0.0001), which is consistent with the tumor suppressor role of PPM1a in liver cancer. Furthermore, it seems that higher PPM1a expression in HCC indicates a worse TNM stage (Figure 5B). Last but not the least, although there was no significant difference in HBx staining intensity between HCC and pericarcinous tissue (P=0.6364), our data revealed the negative correlation between PPM1a and HBx expression intensity in HCC tissue (r = −0.3961, P = 0.0114) (Figure 5B). These results further support the argument that HBx is at least partially responsible for down-regulation of PPM1a in HCC tissue.

## DISCUSSION

HBx is a multifunctional viral protein involved in modulating virus replication and various biological activities of HBV-infected hepatocytes. Regulation on the expression of host or other viral proteins by HBx is the major mechanism for its action. Ubiquitin proteasome system (UPS), responsible for the degradation of a majority of intracellular proteins, is partially involved in this process [27]. HBx can interact with components of the UPS, including the CUL4 adaptor DDB1, the cullin regulatory complex CSN, and the 26S proteasome, and then alter the levels of target proteins [28]. Utilization of ubiquitin proteasome machinery by HBx has become one of the strategies for its promotion on HCC progression. Thus, understanding the functional interactions between HBx and UPS and uncovering their novel targeted proteins would provide new clues for the pathogenesis of HCC.

Our data here showed that HBx enhanced ubiquitination of PPM1a, and subsequently led to the decreased PPM1a protein level in a proteasome-dependent manner. Recently, PPM1a attracts more interests especially for its suppression role in tumorigenesis. Both in bladder cancer and metastatic prostate cancer, expression of PPM1a decreases and negatively correlates with the histological grade, metastasis and survival time of the patients [19, 20]. Animal experiments further proves that PPM1a inhibits metastasis of above cancers [19, 20]. Similarly, our study with human HCC specimens show that PPM1a expression in liver cancer tissue is weakened, further supporting PPM1a as a tumor suppressor. Furthermore, we showed that HBx enhanced the ubiquitination of PPM1a and accelerated its degradation in a proteasome-dependent manner, which is the first report about the regulatory mechanism of PPM1a. Our findings seem to be consistent with the previous report that HBx dose-dependently reduced enzyme activity of recombinant PP2Cα/PPM1a and suppressed PPM1a-mediated effects on cell survival. However, as HBx is able to interact with several components of the UPS, we are not sure about the conclusive molecular mechanism how HBx enhances ubiquitination of PPM1a. Further studies are needed.

It has been proven that PPM1a is the phosphatase responsible for dephosphorylating p-Smad2/3, thus playing an important role in terminating TGF-β signaling pathway [17]. Upon TGF-β stimulation, PPM1a level is upregulated, suggesting the feedback between TGF-β and PPM1a [29]. Our study here clearly showed that HBx destroyed this feedback. This interference of HBx on the feedback greatly amplify and prolong effects of TGF-β signal, which are important for HCC development.

However, the inhibition of PPM1a seems to be TGF-β-dependent. In previous studies, PPM1a down-regulation leads to a dramatic promotion on migration and invasiveness of bladder cancer, while inhibitors of TβRI (SB431542) treatment markedly abolished this effect [20]. Here, we found that only upon TGF-β stimulation, HBx significantly increased the level of p-Smad2/3, coincident with downregulated PPM1a expression. That might be the reason why the decrease of PPM1a occur only in HCC tissue rather than in pericarcinous tissue as the amount of TGF-β in HCC tissue is much higher [23, 24]. These results would offer a novel explanation for over-activation of TGF-β signaling pathway in HBV-related HCC.

**Figure 6:**
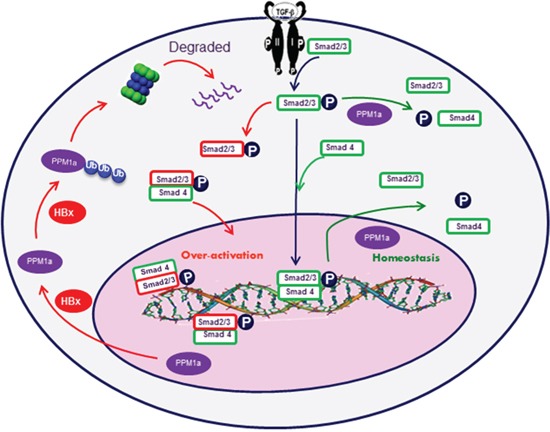
Graphic model as discussed in the text After TGF-β binding to its cell surface receptors, Smad2 and Smad3 are phosphorylated in the cytoplasm. Phosphorylated Smad2 and Smad3 are then transported into the nucleus with the help of Smad4, where they cooperate with specific transcription factors and regulate gene transcription. Feedback upregulation of PPM1a dephosphorylate p-Smad2/3 and terminates the signal pathway to maintain cellular homeostasis. However, in HBx-related HCC cells, HBx promotes the ubiquitin-mediated degradation of PPM1a and over-activates TGF-β signal pathway, which fosters HCC metastasis.

In summary, in the present study, we showed that HBx increases PPM1a ubiquitination and accelerates its proteasomal degradation on stimulation of TGF-β. Moreover, HBx-mediated downregulation of PPM1a contributes to HCC migration and invasion (Figure 6). These findings not only substantiate the critical role of HBx in promotion of TGF-β signaling pathway, but also uncover a novel mechanism for HBx involvement in HCC progression, which would shed new light on discovering the potential candidates for HCC prognosis and treatment.

## MATERIALS AND METHODS

### Cell lines and cultures

HepG2 and Bel-7402 cells, two human HCC cell lines, were maintained in DMEM supplemented with 10% fetal bovine serum (FBS) at 37°C in an incubator containing 5% CO2. Cells at 70%~90% density were transfected with expression plasmids or empty vectors using Lipofect-amine2000™ reagent (Invitrogen). 5ng/ml of TGF-β was added to cell culture as described before [27].

### Reagents

Rabbit anti-PPM1a antibody, mouse anti-HA antibody, rabbit anti-pSmad3 antibody and mouse anti-HBx antibody were purchased from Abcam (Cambridge, MA). Mouse anti-ubiquitin antibody was purchased from Santa Cruz (Santa Cruz, CA). Human TGF-βwas purchased from obtained from R&D Systems (Minneapolis, MN).

### Transwell assays

Transwell chambers (polycarbonate filters of 8 μm porosity, Millipore) were used for transwell assay. 1 × 10^5^ cells, suspended in serum-free media, were seeded in the upper chamber. Culture medium supplemented with 10% FBS was added into the bottom chamber. After incubation for a period of time (10h for migration and 24h for invasion), migrated cells were fixed with 100% ethanol for 30 minutes and stained with 0.1% crystal violet for 20 minutes. Stained cells were visualized by microscope.

### Tissue microarrays

Tissue microarrays were purchased from Outdo biotech (Shanghai, China). Single HCC and matched pericarcinous tissue cores (2 mm in diameter) were sampled from 41 HCC patients and assembled into a recipient paraffin block using a TMA instrument. Detailed information of the patients was provided in Supplementary Table 1.

### Immunohistochemistry staining assay

4-μm formalin-fixed paraffin-embedded (FFPE) sections were used for immunohistochemical staining analyses. The sections were deparaffinized, rehydrated and incubated in EDTA at 120°C for 5 min to retrieve the antigen. After treatment with 3% H_2_O_2_ at 37°C for 30 minutes, tissue were blocked with goat serum for 30 minutes and incubated with primary antibodies overnight. Primary antibodies, as described before, were diluted according to the instructions. Sections were then incubated with peroxidase-labeled polymer conjugated to goat anti-mouse or anti-rabbit immunoglobulin G (IgG) (ZSJQ-Bio, China). Finally, sections were developed with 3,3′-diaminobenzidine tetrahydrochloride (DAB;ZSJQ-Bio, China). Hematoxylin was used for counterstaining. The immunohistochemical staining was evaluated by two experienced pathologists independently. The average intensity was given a score from 0 to 3 corresponding to the presence of negative, weak, intermediate, and strong staining, respectively. The intensity score was multiplied by the extent of the staining (%) to form a final score. The specific method for HBx staining has been reported before [30].

### RT-PCR and real-time PCR

Total RNA was isolated using the Trizol, precipitated by isopropyl alcohol and washed with 70% ethanol. The cDNA was synthesized from 1ug total RNA by reverse transcription, using an cDNA synthesis kit. PCR cycling procedures were as follows: 26–35 (RT-PCR) or 40 (Real-time PCR) cycles of denaturation at 95°C for 30 seconds, followed by annealing at 56–60°C for 30 seconds and extension at 72°C for 30 seconds. The specific primer sequences were as follows. HBx: (F:5′-TCCTTTGTCTACGTCCCG; R:5′-TAATCTCCTCCCCCAACTCCTC-3′), PPM1a: (F5′-AGGGGCAGGGTAATGGGTT-3′;R:5′-GATCACAGCCGTATGTGCATC-3′), Actin: (F:5′-AGTTGCGTTACACCCTTTC-3′;R:5′-CCTTCACCGTTCCAGTTT-3′). The RT-PCR products were analyzed using agarose gel electrophoresis.

### Western blot

Cells were harvested and lysed by cell lysis buffer. Total cell lysates were separated by 10%SDS PAGE and transferred to nitrocellulose membranes after electrophoresis. Then the membranes were blocked with 3% BSA for 3 hours and incubated with various antibodies (1:1000) overnight at 4°C. The membranes were then incubated in HRP-conjugated anti-mouse IgG or anti-rabbit IgG Abs (diluted 1/5000 in 3%BSA) for 2 h and a chemiluminescent substrate (ECL, Amersham Biosciences, UK) was used to detect the immunoreactive bands.

### Immunoprecipitation

Cells were harvested and lysed with RIPA buffer. Cell lysates were incubated with anti-PPM1a Ab at 4°C on a rotation rack for 2 hours, then A/G PLUS-Agarose beads was added and the mixture was maintained on the rotation rack at 4°C for another 8 hours. After washing and centrifugation for 5 times, immune complexes were collected and western blot assay was performed with an anti-ubiquitin antibody.

### Statistical analysis

Statistical analysis was performed using GraphPad Prism 5 and SPSS 18.0. Differences between two groups were assessed by using Student's t test. The correlation between variables was analyzed by using Spearman rank correlation coefficient test. P < 0.05 was regarded as statistically significant.

## SUPPLEMENTARY FIGURES AND TABLES


