# Supplementary material for: Hepatitis B virus X protein amplifies TGF-β promotion on HCC motility through down-regulating PPM1a
Source: Oncotarget. 2016 Apr 21;7(22):33125–35. doi: 10.18632/oncotarget.8884 (PMC5078080; doi:10.18632/oncotarget.8884)
Supplement: Supplementary file 1 [file oncotarget-07-33125-s001.pdf]

SUPPLEMENTARY FIGURES AND TABLE

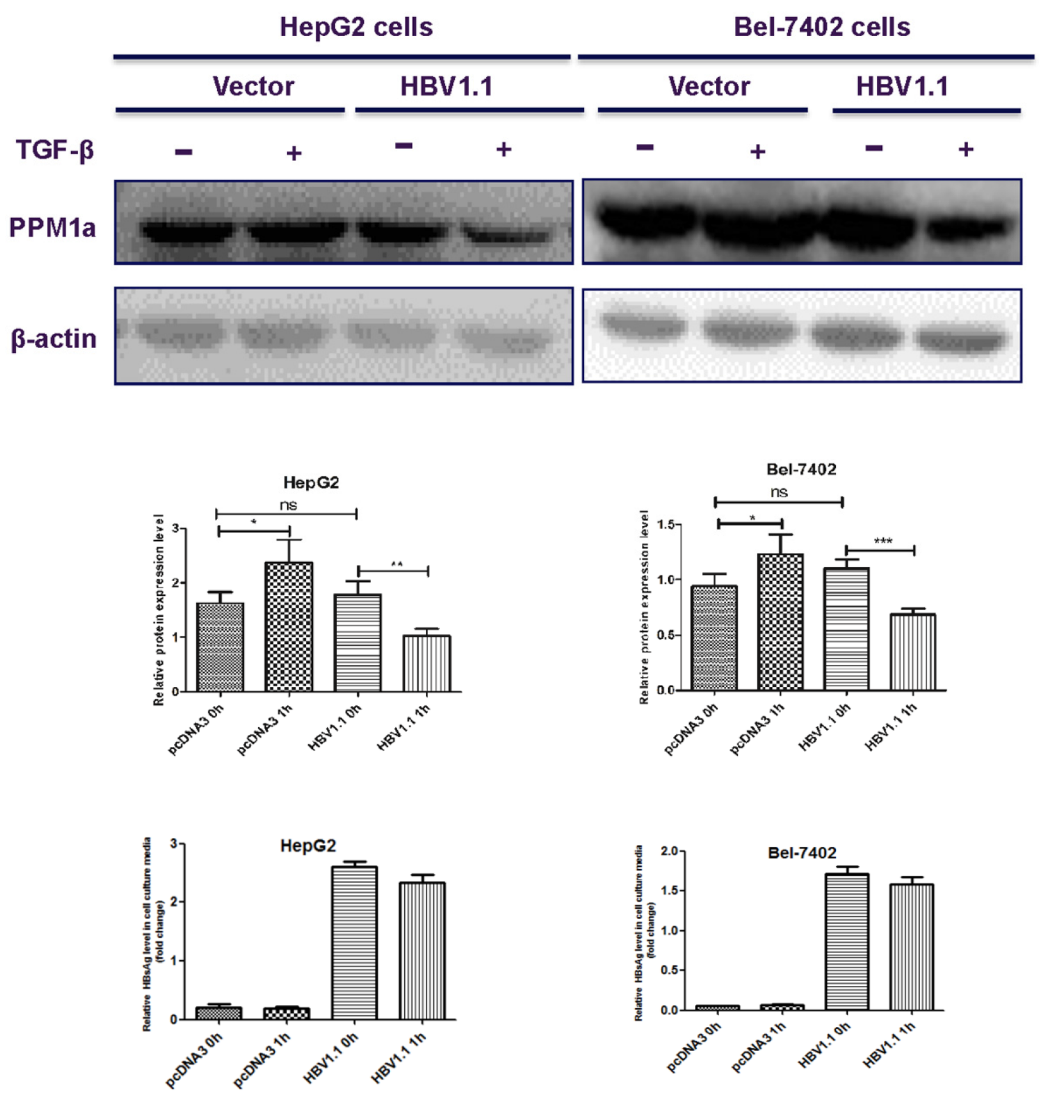

Supplementary Figure S1: HBV downregulated PPM1a expression in the presence of TGF-β. HepG2 and Bel-7402 cells were transfected with pcDNA3-HBV1.1 or pcDNA3 vector and then stimulated with TGF-β or PBS for 1h. Expression of PPM1a measured by Western blot(upper panel). Band intensity of each Western Blot image is analyzed by the software Image J(middle panel). HBsAg in cell culture media was detected by Elisa to make sure that transfection was succeeded (low panel).

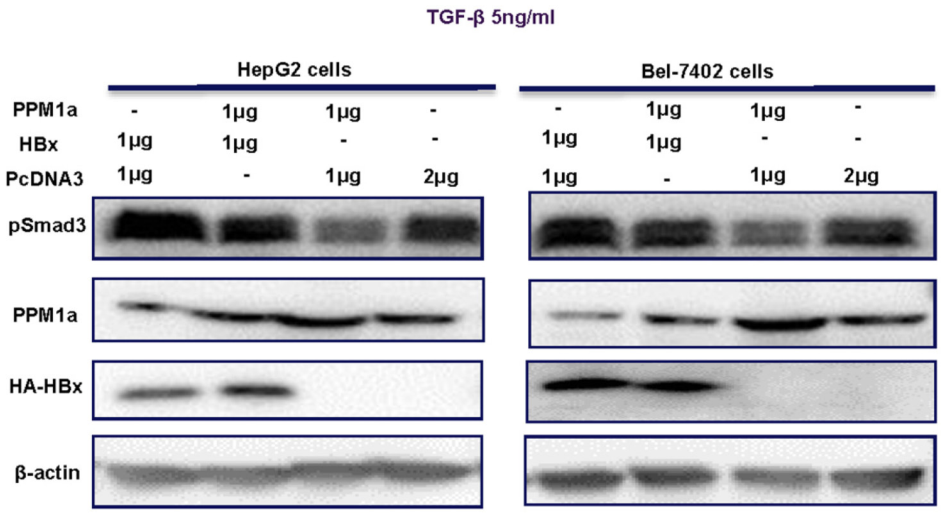

Supplementary Figure S2: PPM1a expression in plasmid rescue experiment. Transfection of 1μg of pcDNA3-PPM1a-Flag almost leveled PPM1a expression of HBx-transfected cells to that of control cells.

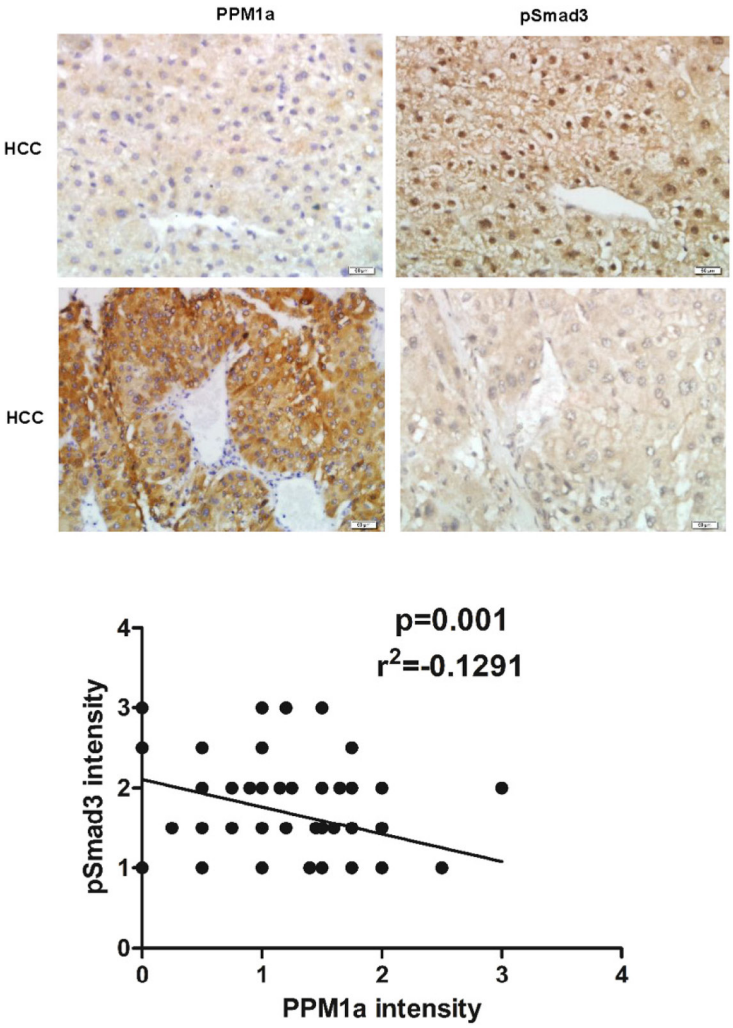

Supplementary Figure S3: Negative correlation between pSmad3 and PPM1a expression. Expression of PPM1a and pSmad3 level in HCC tissue were assessed by IHC. Original magnification 400× (upper panel). Correlation between PPM1a and HBx expression intensity were analyzed statistically (low panel).

Supplementary Table S1: Clinicopathologic features of the HCC patients and data regarding HBV/HCV infection (n = 41)

Patient	Sex	Age	tumor stage	tumor stage2	HBcAb	HBeAb	HBeAg	HBsAb	HBsAg	HBVDNA	HCVRNA
**1**	M	48	T3N0M0	II	+	+	-	-	+		-
**2**	M	55	T3N0M0	II-III	+	-	+	-	+		-
**3**	M	49	T3N0M0	II	+	+	-	-	+		-
**4**	M	44	T2N0M0	II	+	-	+	-	+	4.2*10^6	-
**5**	M	61	T3N0M0	III	+	+	-	-	-		-
**6**	M	42	T3N0M0	III	+	+	-	+	+	-	-
**7**	M	62	T2N0M0	II	+	-	+	-	+	5.88*10^4	-
**8**	F	43	T2N0M0	I	+	-	+	-	+	8.26*10^5	-
**9**	M	43	T1N0M0	II	+	+	-	-	+	4.07*10^6	-
**10**	M	45	T2N0M0	III-IV	+	+	-	-	+	4.4*10^4	-
**11**	M	68	T2NxM0	III	-	-	-	-	-	-	-
**12**	M	54	T2NxM0	II	-	-	-	-	-	-	-
**13**	M	39	T2NxM0	II-III							
**14**	M	47	T1N0M0	II	+	+	-	-	+	-	-
**15**	F	37		II-III					+		
**16**	M	47		II							
**17**	M	48		II-III	-	+	-	-	+	-	-
**18**	M	42	T4NxMx	II-III							
**19**	M	34		II-III							
**20**	M	42	T3NxM0	II	+	-	-	-	+	+	+
**21**	M	54	T1N0M0	II	-	+	-	-	+		
**22**	M	35		II							
**23**	M	45		III	+	-	-	-	+	+	-
**24**	M	53		II	-	-	-	-	-	-	-
**25**	M	68		III	+	-	+	-	+	+	-
**26**	M	37		II	+	+	-	-	+	-	-
**27**	M	53	T2N0M0	II	-	-	-	-	+	-	-
**28**	F	44	T2N0M0	III	-	-	-	-	+	-	-
**29**	F	63		II	+	+	-	-	+	-	-
**30**	M	48		II							
**31**	M	54	T2N0Mx	II-III	+	+	-	-	+	+	-
**32**	M	59		II	+	+	-	-	+	-	-
**33**	M	40	T2N0Mx	II	+	+	-	-	+	-	-
**34**	F	52	T4NxM0	II	-	+	-	-	+	-	-
**35**	M	53	T3NxM0	II	-	-	-	-	+		
**36**	M	54	T2N0M0	II	+	-	+	-	+		
**37**	F	60	T2N0M0	II	+	+	-	+	+	-	+
**38**	M	52		II-III	-	-	-	+	-		
**39**	M	44	T3NxM0	II	+	+	-	-	+	-	-
**40**	M	65		II	+	+	-	-	+		
**41**	M	61	T1NxM0	II	-	-	-	+	-	-	-
